# Commentary: Toward a more physiologically and evolutionarily relevant definition of metal hyperaccumulation in plants

**DOI:** 10.3389/fpls.2015.00554

**Published:** 2015-07-22

**Authors:** Antony van der Ent, Alan J. M. Baker, Roger D. Reeves, A. Joseph Pollard, Henk Schat

**Affiliations:** ^1^Centre for Mined Land Rehabilitation, Sustainable Minerals Institute, The University of QueenslandBrisbane, QLD, Australia; ^2^Laboratoire Sols et Environnement, Université de Lorraine - INRA, UMR 1120Nancy, France; ^3^School of BioSciences, The University of MelbourneParkville, VIC, Australia; ^4^Massey UniversityPalmerston North, New Zealand; ^5^Department of Biology, Furman UniversityGreenville SC, USA; ^6^Department of Genetics, Faculty of Earth and Life Sciences, Vrije Universiteit AmsterdamAmsterdam, Netherlands

**Keywords:** excluder, hyperaccumulator, metallophyte, metal-tolerance, spiked soils

## Introduction

In our review (Van der Ent et al., 2013) we attempted to clarify the definition of hyperaccumulation of trace elements, to explain the relevant ecophysiological considerations, and to identify points of confusion, which had systematically occurred in many publications. We feel that the Opinion article in *Frontiers in Plant Science* by Goolsby and Mason (2015) introduces a number of important misunderstandings that challenge our basic tenets and therefore require examination.

## Evolution of hyperaccumulator plants in natural and anthropogenic systems

Contrary to the statement by Goolsby and Mason, anthropogenic sources have not given rise to new hyperaccumulator plants. There is sound evidence that hyperaccumulator species now frequently associated with anthropogenic sources of soil contamination (e.g., *Arabidopsis halleri* and *Noccaea caerulescens*) acquired this property in the course of their evolution millions of years ago (Pauwels et al., 2006; Hanikenne and Nouet, 2011). Indeed, the primary habitats of most hyperaccumulator plants are pristine geogenic sites such as serpentine outcrops, where exposure to surface mineralization results from prehistoric geological processes (Baker et al., 2010). We have never claimed that all populations of hyperaccumulator plants, however, are necessarily restricted to metalliferous environments, as demonstrated by at least 36 known taxa that are facultative hyperaccumulators, occurring on both normal and metalliferous soils (Pollard et al., 2014).

## Allegations of hyperaccumulation in crops and other species of non-metalliferous soils

No domesticated crop meet the stringent condition that the complete life cycle must be observed through to production of viable seed while having foliar concentrations in excess of threshold levels. Many plant species can be made to seemingly “hyperaccumulate” trace elements by exposure to exceedingly high metal concentrations, but this results from breakdown of metal homeostasis mechanisms and is followed by obvious toxicity symptoms and death (Baker, 1981). On the other hand, the idea that some hyperaccumulator plants may display “inadvertent uptake” based on traits that were not evolved on metalliferous soils is not new (Boyd and Martens, 1992). For example, *Phytolacca americana* has over 1% Mn in its leaves on Mn mines in both China and the USA (Pollard et al., 2009). Data from both countries indicate that the ability to hyperaccumulate exists in most or all populations, but it has been suggested that this is a side effect of acidic root exudates evolved as a P-acquisition mechanism (Lambers et al., 2015). In some cases it may be appropriate to study metal uptake in species from non-metalliferous soils, but only when driven by specific hypotheses such as comparisons among conspecific metallophytes and non-metallophytes in a primarily hyperaccumulating lineage such as *Alyssum* sect. *Odontarrhena*.

## The use of spiked soils in studying hyperaccumulator plants

Although a minimum trace element concentration is needed in the soil to achieve uptake, the key characteristic of hyperaccumulator plants is their highly efficient uptake behavior and a non-linear response to soil trace element concentrations (Baker, 1981). For example, *N. caerulescens* achieves >1% foliar Zn when growing on soils with only background concentrations of Zn (Reeves et al., 2001). There is no evidence that pot-grown hyperaccumulator plants quickly exhaust the available metals in the soil in most experimental settings, as alleged by Goolsby and Mason. Conversely, there are major issues with metal availability equilibration in spiked soils and it is clear from isotope studies that soil contains different trace element pools in a dynamic equilibrium (Echevarria et al., 1998). Soil trace element-bearing phases have different time scales for interchange and release of trace elements, and therefore when soluble forms of a trace element are “spiked” it is hard to predict into what phases the metal will load. Goolsby and Mason state “several sources have suggested that using artificially amended or ‘spiked’ soils in greenhouse studies of hyperaccumulation are undesirable for both identifying hyperaccumulator plants and understanding their physiology.” Although we have argued that such methods are insufficient for identifying hyperaccumulator plants, we have never disputed their usefulness in physiological, evolutionary and genetic experiments. For example, to investigate the ecological potential of a species or ecotype it may be necessary to explore the limits of its homeostasis by exposure to manipulations such as spiked soils or nutrient solutions. However, we wish to emphasize that the rhizosphere biotic system is critical for the normal ecophysiological performance of a plant and when grown in the absence of this component, the system is totally artificial and unrepresentative of its natural environment. For example, the Ni hyperaccumulator *Berkheya coddii* is strongly mycorrhizal and has poor survival and Ni uptake without this association (Orłowska et al., 2011).

## Genetic traits of metal-tolerance and metal-hyperaccumulation

Hyperaccumulator plants, even in their non-metallicolous populations (Pauwels et al., 2006), are extremely tolerant to the element accumulated. However, metal tolerance and metal hyperaccumulation are genetically separable, at least to some degree (Macnair et al., 1999; Macnair, 2003; Richau and Schat, 2009), although there are common determinants, such as HMA4 in *A. halleri* (Courbot et al., 2007; Hanikenne et al., 2008). Typically, on any metalliferous soil, there are many more species that are tolerant without hyperaccumulating; the majority are metal excluders (Baker, 1987). Is there really a near impossibility of disentangling tolerance and accumulation if only naturally-occurring hyperaccumulator plants are used? The studies cited above, and many others over the last two decades, have made substantial progress in doing just that. Evolution through natural selection, by definition, has taken place in nature on natural soil, not in a greenhouse in artificially spiked soils. Although the conditions under which the trait has been evolved remain uncertain, natural hyperaccumulation in a present-day population is indicative of ecological relevance, in contrast to induced hyperaccumulation from spiked soils or nutrient solutions. If there is no ecological function or advantage, there is no demonstrable evolutionary relevance, because natural selection can only act on traits that are expressed in nature, not on “artificially-inducible phenotypes.” Extreme natural phenotypes such as hyperaccumulators must have evolved through positive, directional natural selection, rather than neutral genetic drift. Artificially-induced phenotypes are often manifestations of “stress” caused by disruption of the plant's homeostasis under extreme conditions. It is unthinkable that extreme, but consistent, phenotypes in nature, such as hyperaccumulator plants, would be a manifestation of a continuously disturbed homeostasis. On the contrary, such phenotypes reflect genetic adaptation.

## Recognition of hyperaccumulator plants on the basis of natural populations

Fundamentally, hyperaccumulation as observed in nature results from the interaction between plant species and their environments. Our definitions, based on elemental concentration criteria, are phenomenological, relating to observable properties of plants in the field and justified by the orders-of-magnitude differences in concentrations observed between certain plant species and (i) most others growing in the same soil environment, and (ii) most others growing on less metalliferous soils. These concentration-based definitions serve the very useful practical purpose of drawing attention to a relatively rare plant response to particular elements. Identification of hyperaccumulator behavior in the natural environment can then lead to studies on plant physiology and molecular biology, and to practical applications in phytoextractive technologies. Next-generation sequencing can lead to a better understanding of the evolutionary path toward hyperaccumulation and extend knowledge to species other than model plants (Verbruggen et al., 2012). Comparisons of hyperaccumulator plants with closely related non-accumulator species (where these exist) are especially fruitful. Far from having no relevance to evolution, virtually all of what we presently know about hyperaccumulator physiology and evolution is based on studies using species that have been selected on the basis of their foliar metal concentrations in present-day natural populations.

### Conflict of interest statement

The authors declare that the research was conducted in the absence of any commercial or financial relationships that could be construed as a potential conflict of interest.
